# Delayed Effects of Acute Reperfusion on Vascular Remodeling and Late-Phase Functional Recovery After Stroke

**DOI:** 10.3389/fnins.2019.00767

**Published:** 2019-07-23

**Authors:** Violeta Durán-Laforet, David Fernández-López, Alicia García-Culebras, Juan González-Hijón, Ana Moraga, Sara Palma-Tortosa, Isaac García-Yébenes, Adrián Vega-Pérez, Ignacio Lizasoain, María Ángeles Moro

**Affiliations:** Unidad de Investigación Neurovascular, Departamento de Farmacología y Toxicología, Facultad de Medicina, Instituto Universitario de Investigación en Neuroquímica, Universidad Complutense de Madrid, Instituto de Investigación Hospital 12 de Octubre (i+12), Madrid, Spain

**Keywords:** brain, recanalization, perfusion, blood-brain barrier, angiogenesis, stroke

## Abstract

Tissue perfusion is a necessary condition for vessel survival that can be compromised under ischemic conditions. Following stroke, delayed effects of early brain reperfusion on the vascular substrate necessary for remodeling, perfusion and maintenance of proper peri-lesional hemodynamics are unknown. Such aspects of ischemic injury progression may be critical for neurological recovery in stroke patients. This study aims to describe the impact of early, non-thrombolytic reperfusion on the vascular brain component and its potential contribution to tissue remodeling and long-term functional recovery beyond the acute phase after stroke in 3-month-old male C57bl/6 mice. Permanent (pMCAO) and transient (60 min, tMCAO) brain ischemia mouse models were used for characterizing the effect of early, non-thrombolytic reperfusion on the brain vasculature. Analysis of different vascular parameters (vessel density, proliferation, degeneration and perfusion) revealed that, while early middle cerebral artery recanalization was not sufficient to prevent sub-acute vascular degeneration within the ischemic brain regions, brain reperfusion promoted a secondary wave of vascular remodeling in the peri-lesional regions, which led to improved perfusion of the ischemic boundaries and late-phase neurological recovery. This study concluded that acute, non-thrombolytic artery recanalization following stroke favors late-phase vascular remodeling and improves peri-lesional perfusion, contributing to secondary functional recovery.

## Introduction

Long-term, stroke-induced vascular remodeling is a key process governing neural tissue reorganization in response to injury (Navarro-Sobrino et al., 2011; Chen et al., 2014). Local tissue perfusion, hemodynamics and proper neurovascular coupling in the peri-lesional regions contribute to support the metabolic demands necessary for neuronal plasticity and for the survival and differentiation of migrating neural progenitors (Thored et al., 2007; Snapyan et al., 2009), two processes that may underlie long-term functional recovery. Indeed, a functional correlation exists between delayed blood flow restoration and neurological recovery in stroke patients (Krupinski et al., 1993; Chopp et al., 2007; Arkuszewski et al., 2009; Navarro-Sobrino et al., 2011). Additionally, in the context of vascular remodeling upon damage, vascular perfusion is necessary for vessel survival, stimulates vessel outgrowth and precludes endothelial degeneration (Dimmeler et al., 1996; Haga et al., 2003; Yamamoto et al., 2003). Thus, acute artery recanalization may condition the long-term survival of a vascular substrate for remodeling and vascularization of the ischemic and peri-ischemic brain regions during the chronic neural repair phase after stroke. Several studies have explored vascular remodeling after stroke (Marti et al., 2000; Wei et al., 2001; Zhang et al., 2002; Hayashi et al., 2003; Schaffer et al., 2006; Mostany et al., 2010; Hayakawa et al., 2012; Mao et al., 2014; Marks et al., 2014). In the present report, we intend to go further and elucidate the implication of vessel reperfusion after brain ischemia and also to compare it with a non-reperfusion scenario.

Early restoration of brain tissue perfusion is the main strategy currently pursued in the acute management of stroke patients. However, between 17 and 24% of ischemic stroke patients experience spontaneous arterial recanalization within the first 24 h after stroke onset in the absence of induced thrombolysis (Kassem-Moussa and Graffagnino, 2002; Rha and Saver, 2007). Adding to this, the number of patients undergoing recanalization induced by mechanical thrombectomy or thrombus removal in the absence of thrombolysis is increasing (Smith et al., 2017). In spite of its relative frequency, clinical studies describing the impact of non-thrombolytic reperfusion on the long-term progression of the brain vascular component are scarce. In this study, we have characterized for the first time the effect of arterial recanalization on key components of sub-acute (24h-5d) vascular responses and long-term (7d-14d) vascular remodeling both in the ischemic and the peri-lesional brain regions, focusing on critical aspects for late-phase recovery such as local tissue perfusion, blood-brain barrier functionality and permissiveness of the glial scar for remodeling of the brain vascular network in C57bl/6 mice.

## Results

### Early Reperfusion Is Not Sufficient to Prevent Sub-Acute Vessel Degeneration After Stroke

**FIGURE 1:**
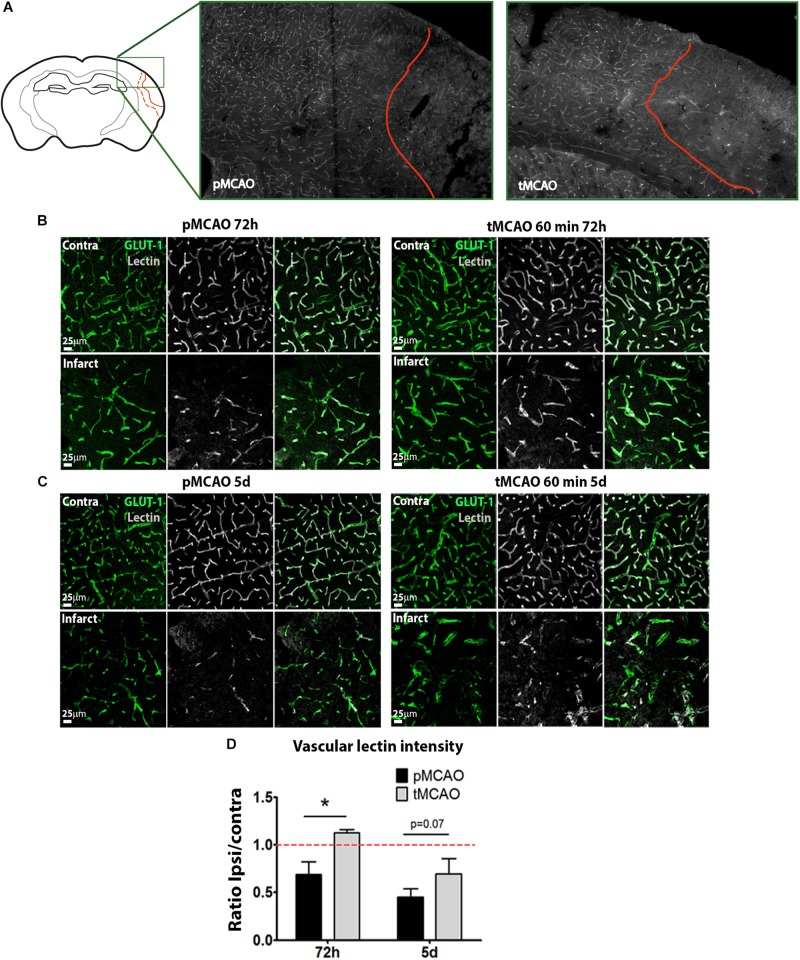
**(A)** Representative images of the whole area of study comprising the healthy tissue, the peri-infarct and the infarct. **(B,C)** Representative confocal fluorescence micrographs (20x) showing perfusion intensity in ischemic and contralateral regions at 72 h **(B)** and 5 days **(C)** after pMCAO or 60-min tMCAO. Intravascular perfusion (lectin, gray) was analyzed in Glut-1^+^ vessels (green). **(D)** Quantification of average lectin intensity in vessels expressed as ratio of ischemic/contralateral (internal control) sub-acutely (*n* = 6–7). ^*^ Student’s *t*-test *p* < 0.05 vs. pMCAO.

**FIGURE 2:**
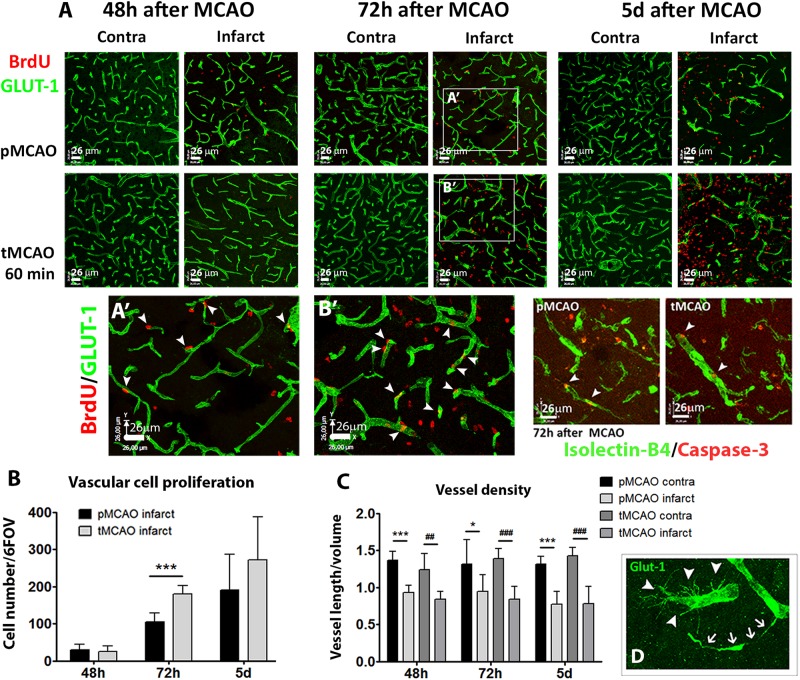
**(A)** Representative confocal fluorescencemicrographs (20x) showing Glut-1^+^ vessels (green) and proliferating BrdU^+^ cells (red), or isolectin-B4^+^ vessels (green) and activated caspase-3^+^ cells (red) at several sub-acute time points after pMCAO or 60-min tMCAO. **(A′,B′)** show magnified fields of view of Glut-1/BrdU immunofluorescence in the infarction at 72 h after MCAO. **(B)** Quantification of vascular cell proliferation expressed as sum of cells in 6 fields of view in the infarction (*n* = 4–7). **(C)** Quantification of vessel density expressed as the ratio of the sum of vessel length by volume unit in six fields of view per region and animal (*n* = 4–7). **(D)** Representative magnified view showing simultaneous presence of angiogenic (arrowheads) and degenerating (arrows) Glut-1^+^ vessels. ^∗∗∗^Student’s *t*-test *p* < 0.001 vs. pMCAO **(B)**; ^∗∗∗^Student’s *t*-test *p* < 0.001 vs. pMCAO contra **(C)**; ##, ###Student’s *t*-test *p* < 0.01, *p* < 0.001 vs. tMCAO contra **(C)**.

The presence of perfused vessels in the sub-acute phase was measured within the infarction at 72 h and 5 days after stroke by quantification of intravascular FITC-lectin intensity. Symmetric fields of view in the contralateral cortex were used as internal controls. The tMCAO procedure led to increased sub-acute perfused vessels irrigating the infarction at 72 h (*p* < 0.05, Figures 1B,D) and 5 days (*p* = 0.07, Figures 1C,D) as compared to pMCAO. The impact of early reperfusion on the vascular niche was characterized within the infarction by two different approaches: 1) quantification of vascular cell proliferation as a measure of stroke-induced angiogenesis and 2) quantification of vessel density as end-point parameter for vascular remodeling. Vascular cell proliferation was analyzed cumulatively by combining several protocols of BrdU administration (Supplementary Figure 2A). Following permanent artery occlusion (pMCAO group), vascular proliferation labeled from 24h after stroke was evident from 48h up to the latest time studied, 5 days after stroke (Figures 2A,A′ arrowheads, B). When measured at 72 h, early reperfusion led to increased cumulative vascular cell proliferation (Figures 2B,B′ arrowheads, B), suggesting an enhancement of the proliferative vascular response to stroke in presence of reperfusion. However, cumulative proliferating cell numbers were similar between pMCAO and tMCAO groups at 5 days (*p* > 0.05, Figures 2A,B). Additionally, vessel density decreased after both pMCAO and tMCAO at 48 h up to 5 days after stroke and was not affected by differences in vascular cell proliferation (Figures 2A,C), suggesting that proliferation could be counteracted by vascular degeneration, regardless of the presence of reperfusion. This was supported by the occasional presence of vessels with recognizable tip cells showing a crown of extended filopodia, a characteristic feature of angiogenesis (Figure 2D, arrowheads) next to interrupted vessels with narrowed lumen, a typical feature of vascular degeneration (Figure 2D, arrows). Vessel degeneration was further revealed by identification of endothelial cells undergoing active apoptosis after both pMCAO and tMCAO (Figure 2A, Isolectin-B4^+^/caspase-3^+^). Together, these observations indicate that improved local measurement of perfused vessels and increased vascular cell proliferation achieved by early reperfusion are not sufficient to prevent sub-acute vessel degeneration within the infarction.

### Acute Reperfusion Favors Peri-Lesional Vascular Remodeling Two Weeks After Stroke

**FIGURE 3:**
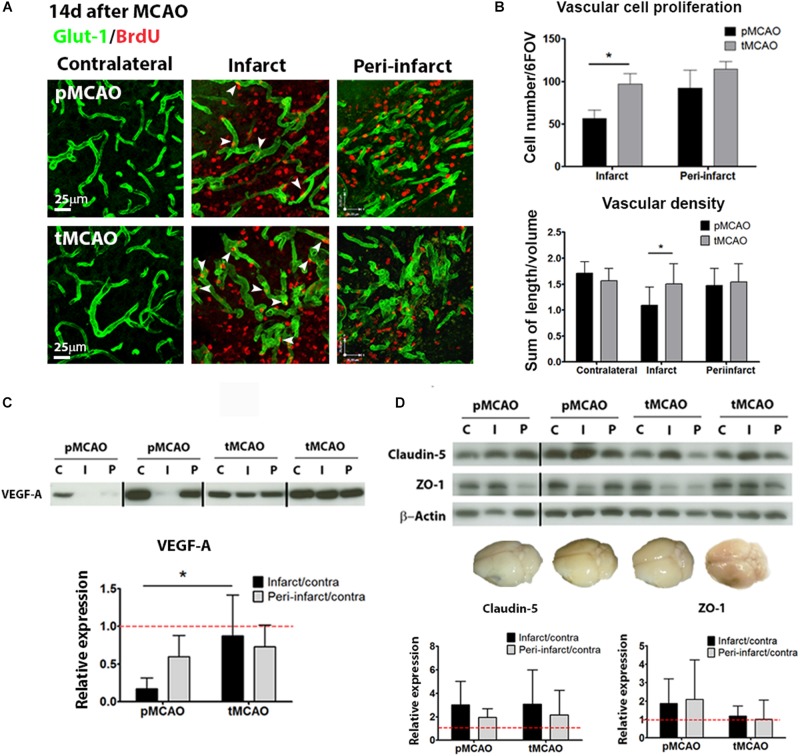
**(A)** Representative confocal fluorescence micrographs (40x) showing Glut-1^+^ vessels (green) and proliferating BrdU^+^ cells (red) at post-stroke day 14. **(B)** Quantification of vascular cell proliferation (*n* = 6–8) and vascular density (*n* = 7–9) at post-stroke day 14. **(C)** Cropped blots from a representative VEGF-A western blot and quantification in contralateral (C), infarcted (I) and peri-lesional (P) tissue at post-stroke day 14. Complete gels are shown in Supplementary Figure 3 (*n* = 5–6). **(D)** Cropped blots from representative ZO-1 (*n* = 5–6) and claudin-5 (*n* = 4–6) western blots and quantifications, and brain photographs showing absence of *in vivo* BBB permeability at post-stroke day 14. Complete gels are shown in Supplementary Figure 3. ^*^ Student’s *t*-test *p* < 0.05 vs. pMCAO.

The effect of acute reperfusion on long-term vascular remodeling was studied within the infarcted and peri-lesional regions 2 weeks after stroke (regions of interest and BrdU administration protocol are shown in Supplementary Figures 2B,C, respectively). Reperfusion achieved by tMCAO led to increased vascular cell proliferation within the infarction between post-stroke days 7 and 10 (*p* < 0.05, Figures 3A,B), corresponding with the days of BrdU administration (Supplementary Figure 2B). Increased proliferation was reflected in higher vessel density quantified at post-stroke day 14, as compared to pMCAO (*p* < 0.05, Figures 3A,B). Additionally, protein expression of the pro-angiogenic factor VEGF-A was better preserved within the infarction after tMCAO (*p* < 0.05, Figure 3C). In peri-lesional regions, active vascular cell proliferation was observed to a similar extent after pMCAO and tMCAO (*p* > 0.05, Figure 3B). Also, no differences on vessel density were detected within this region (*p* > 0.05, Figure 3B).

In order to further characterize the functionality of the remodeling vessels at 14 days after injury, structural and functional blood-brain barrier parameters were analyzed. No permeability of the BBB was detected on the Evans Blue *in vivo* permeability assay (representative brain in Figure 3D) regardless of the presence of early reperfusion. Tight junction protein expression (Claudin-5 and ZO-1) was generally preserved within infarcted and peri-lesional regions, also regardless of the presence of early reperfusion (western blots in Figure 3D).

**FIGURE 4:**
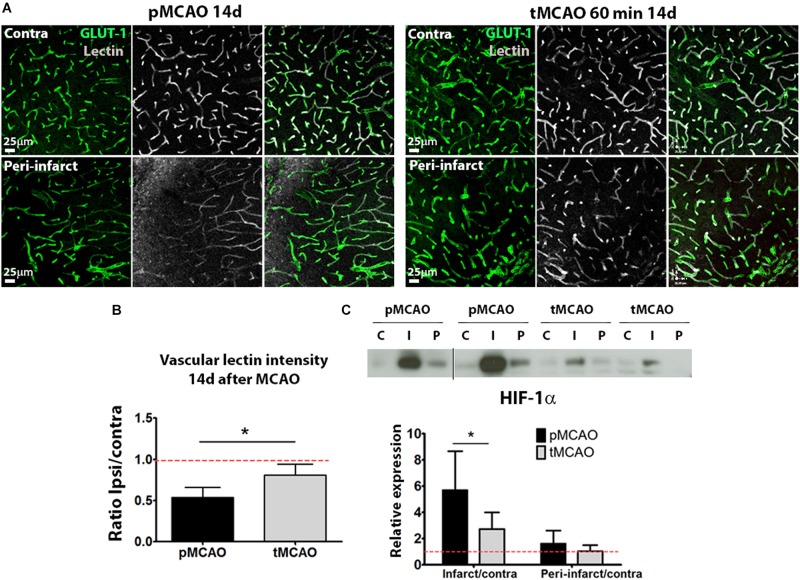
**(A)** Representative confocal fluorescence micrographs (20x) showing perfusion intensity in ischemic and contralateral regions at post-stroke day 14. **(B)** Quantification of average lectin intensity in vessels expressed as ratio of ischemic/contralateral (internal control) sub-acutely. **(C)** Cropped blots from a representative HIF-1α western blot and quantification at post-stroke day 14, expressed as ratio ischemic/contralateral. Complete gels are shown in Supplementary Figure 3 (*n* = 5–6). ^*^Student’s *t*-test *p* < 0.05 vs. pMCAO.

While fluorescent background precluded the quantification of lectin perfusion within the infarction, analysis of vascular lectin intensity within the peri-lesional regions showed that acute recanalization led to a sustained presence of perfused vessels, indicative of local perfusion, in the regions surrounding the injured tissue (*p* < 0.05, Figures 4A,B). Improved peri-lesional perfusion was also reflected in the protein levels of hypoxia-inducible factor-1α (HIF-1α) at 14 days after injury within the infarction, that were significantly lower in animals with acute reperfusion compared to those that underwent permanent ischemia (*p* < 0.05, Figure 4C).

Together, our data indicate that early reperfusion favors long-term peri-lesional perfusion and vascular remodeling within the infarction, while preserving BBB integrity.

### Glial Scar Shows Features of Higher Permissiveness for Brain Tissue Remodeling After Acute Reperfusion

**FIGURE 5:**
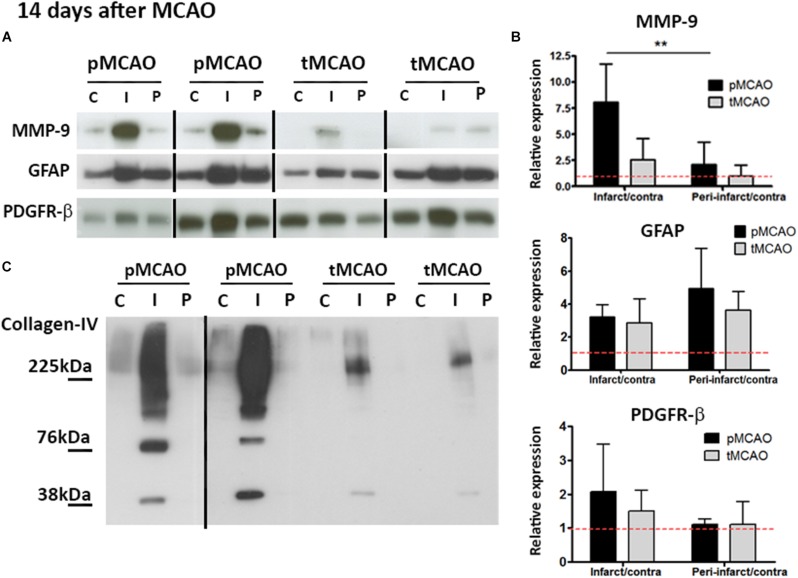
**(A,B)** Cropped blots from representative MMP-9 (*n* = 5–6), GFAP (*n* = 4–5), and PDGFR-β (*n* = 5–6) western blots **(A)** and quantifications **(B)** in contralateral **(C)**, infarcted (I) and peri-lesional (P) regions at post-stroke day 14. ^∗∗^Student’s *t*-test *p* < 0.01 vs. infarct/contra. Complete gels are shown in Supplementary Figure 3. **(C)** Representative collagen-IV cropped western blots showing expression of non-degraded protein (225 kDa) and degradation fragments (76 and 38 kDa) at post-stroke day 14. Complete gels are shown in Supplementary Figure 3.

The glial scar formed in response to CNS injury may preclude tissue repair. Expression of protein markers of two cell populations typically present in the glial scar (GFAP for reactive astrocytes and PDGFR-β for stromal cells of pericytic origin) was quantified at post-stroke day 14 in the infarcted and peri-lesional regions. GFAP expression was expressed to similar extent in both regions after pMCAO and tMCAO (*p* > 0.05, Figures 5A,B). PDGFR-β was also expressed to a similar extent in both stroke models (*p* > 0.05, Figures 5A,B). In contrast, expression of collagen type-IV, a matrix protein abundantly expressed in the scar, as well as of one of its main degrading proteases, MMP-9, were substantially reduced in animals that underwent acute reperfusion as compared to animals with pMCAO (*p* < 0.01, Figures 5A–C). Both intact collagen type-IV (∼230 kDa) and the two main degradation fragments (∼82 kDa and ∼42 kDa) were decreased (Figure 5C), suggesting that decreased levels of intact proteins are not a result of increased degradation and that alteration of matrix composition in the glial scar is a long-term effect of acute reperfusion.

### Acute Reperfusion Favors Late-Phase Functional Recovery

A final question that remained open in the study was if delayed affectation of peri-lesional remodeling mediated by acute reperfusion is associated to on an improvement on long-term injury progression and functional recovery. To address this question, late-phase functional recovery (beyond 48 h after stroke) was characterized in mice after pMCAO and tMCAO.

**FIGURE 6:**
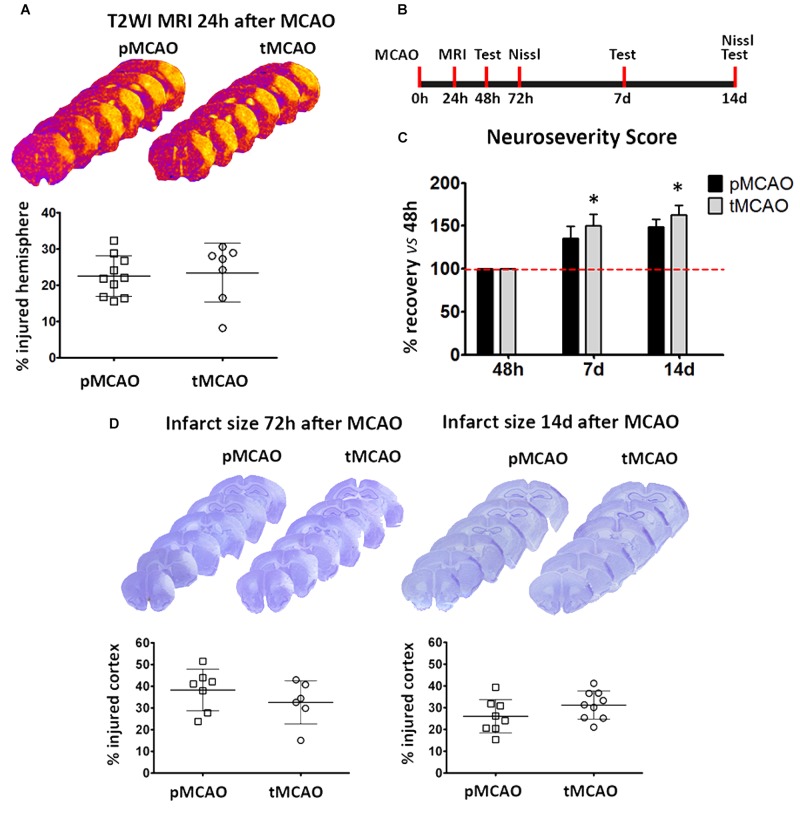
**(A)** Representative series of T2WI-MRI planes and volumetric quantification showing infarct volumes at 24 h after pMCAO or tMCAO. **(B)** Schematic representation of the experimental design for the measurement of infarct volume and functional deficits after MCAO. **(C)** Data from functional assessment performed at 48 h (basal), 7 and 14 days after pMCAO or tMCAO (*n* = 7). **(D)** Representative series of Nissl-stained brain sections and corresponding graphs showing the extent of ischemic injury at 72 h and 14 days after permanent (pMCAO) or transient (60 min) middle cerebral artery occlusion (tMCAO). ^*^Two-way ANOVA *p* < 0.05 vs. pMCAO.

Lesion size at 24 h was similar in mice subjected to pMCAO and 60-min tMCAO (Figures 6A,B). Neurological deficits at 48 h were also comparable between both groups (not shown). In contrast, late-phase recovery from acute deficits (expressed as percentage of recovery versus the deficits observed at 48 h) was significantly higher in mice that underwent early artery recanalization at 7 and 14 days (*p* < 0.05, Figure 6C). However, injury progression (up to 14 days) was similar after pMCAO and tMCAO (*p* > 0.05, Figure 6D), indicating that recovery is not related to a reduction of injury size and could be related to the observed enhancement of vascular function in the peri-lesional regions.

## Discussion

An adequate restoration of tissue perfusion during the first hours after stroke is the main strategic pursued to reduce brain injury. This approach is especially effective when salvageable tissue remains within the ischemic territory. However, it is assumed that stroke-induced injury is permanently established within the first 48 h after stroke regardless of the initial presence of salvageable penumbral tissue (Heiss et al., 1992; Darby et al., 1999; Markus et al., 2004). One of the most interesting findings in this study is that, beyond the known acute effects on brain injury, reperfusion also impacts vascular remodeling and peri-lesional hemodynamics in the long-term. More importantly, delayed effects of acute reperfusion may underlie late-phase functional recovery occurring after injury consolidation. This conclusion extends the clinical importance of achieving early reperfusion for the chronic recovery phase after stroke.

Importantly, as soon as 24 h after stroke there was a significant decrease in vascular coverage within the injured tissue regardless of the presence of tissue reperfusion, despite the fact that vascular degeneration occurred faster after pMCAO than after tMCAO. However, even though reperfusion prolonged vessel survival in the core, eventually restoration of brain perfusion was insufficient to rescue endothelial cells from death caused by ischemia-reperfusion injury. Notably, our data provide new knowledge on the vascular dynamics at the infarct core, an area that remains so far quite understudied.

Relating to this, recent studies have shown that reperfusion is not always complete at the capillary level, since no-reflow phenomenon has been observed in approximately 32% of capillaries within the injured tissue due to sustained constriction of contractile vascular cells, which can in turn trigger capillary degeneration (Yemisci et al., 2009; Hill et al., 2015). Adding to this, reperfusion itself exacerbates endogenous damaging mechanisms that can affect the survival of brain vessels such as oxidative stress and neuroinflammation (Eltzschig and Eckle, 2011; Iadecola and Anrather, 2011), thus increasing blood-brain barrier leakage and vasogenic oedema (Barber et al., 2005; Krueger et al., 2015) and ultimately compromising vessel survival.

The loss of capillary coverage within the infarcted tissue could potentially be compensated by vascular cell proliferation. Consistent with previous reports (Seevinck et al., 2010; Hossmann, 2012), we have observed a peak in vascular cell proliferation between 48 and 72 h after stroke. Interestingly, this peak of proliferation was increased by reperfusion, but was not reflected in higher vascular coverage later on. The simultaneous presence of apoptotic (activated caspase-3^+^) vascular cells observed is in agreement with a prior study (Hayashi et al., 2003), and confirms the co-existence of vascular proliferation and degeneration. Of note, absence of increased vessel density in response to proliferation conflicts with previous reports (Marti et al., 2000; Hayashi et al., 2003), but is a clear observation in our study. Interestingly, our observations at later time points reveal a secondary wave (between post-stroke days 7 and 10) of vascular proliferation within the ischemic and peri-lesional tissue, also described in previous studies (Wei et al., 2001; Zhang et al., 2002). Once more, in spite of the active proliferation and vascular remodeling observed in the peri-lesional regions, vascular coverage remained unchanged regardless of the presence of acute recanalization, in agreement with a previous long-term longitudinal study using intravital microscopy (Mostany et al., 2010). The same study concludes that absence of local angiogenesis does not preclude restoration of cerebral blood flow in the peri-lesional regions. Similarly, improved peri-lesional perfusion that we observed after acute tissue reperfusion can occur in the absence of angiogenesis.

We acknowledge that our study has certain limitations. For instance, transient mechanical vascular occlusion has been suggested not to be a fully adequate model of naturally occurring stroke (Hossmann, 2012) although, in our study, it was deemed appropriate in order to investigate the effect of reperfusion versus the permanent occlusion. In our work, comparisons are established between ipsi- and contralesional sides, thus assuming that the contralesional one does not show any major changes as previously described (Seevinck et al., 2010). However, we cannot discard that minor variations in the contralesional vasculature might take place. In addition, the differential contribution of local (angiogenesis) and/or peripheral (vasculogenesis) endothelial cells/progenitors to vascular outgrowth and remodeling as well as the contribution of recruited collaterals to the improvement of peri-lesional perfusion observed after acute recanalization have not been specifically addressed. It is known that vasculogenesis plays a role in post-stroke vascular remodeling (Hayakawa et al., 2012; Mao et al., 2014), but no correlation with restoration of local cerebral blood flow has been found so far. Regarding collateral circulation, the fact that injury size at 72 h after stroke was similar after pMCAO and tMCAO suggests that compensatory collateral perfusion may occur in the absence of reperfusion, as previously reported (Wei et al., 2001; Schaffer et al., 2006). A clinical study has shown that reperfusion following artery recanalization is a better prognostic marker of good outcome than of good collateral circulation (Marks et al., 2014). Collaterals contribute to improve reperfusion following recanalization (Heiss et al., 1992; Darby et al., 1999; Kassem-Moussa and Graffagnino, 2002; Markus et al., 2004; Barber et al., 2005; Rha and Saver, 2007; Yemisci et al., 2009; Seevinck et al., 2010; Bang et al., 2011; Eltzschig and Eckle, 2011; Iadecola and Anrather, 2011; Hossmann, 2012; Liebeskind et al., 2014; Marks et al., 2014; Hill et al., 2015; Krueger et al., 2015; Smith et al., 2017), but to our knowledge a reverse effect of recanalization on collateral circulation has not been yet described. However, we acknowledge the possibility that leptomeningeal collateral remodeling induced by reperfusion may have contributed to the outcomes described here. As far as functional recovery is concerned, our study explores up to 14 days after MCAO; further studies are required to ascertain whether this effect remains at later time points. Finally, reperfusion in the tMCAO was assessed visually. Although this is a quite straightforward procedure when a craniectomy has been performed, as in our case, we acknowledge that this method lacks the resolution of laser Doppler flowmetry.

Our exploration of the injury scar has revealed interesting effects of acute recanalization, especially in what refers to the extracellular matrix composition and turnover. We focused on characterizing expression of the reactive astrocytes marker GFAP and the pericyte marker PDGFR-β, since recent studies have described that a specific subpopulation of pericytes characterized by the expression of this marker are the main source of stromal cells forming the scar as a result of spinal cord injury (Göritz et al., 2011; Soderblom et al., 2013). While recanalization affected neither GFAP nor PDGR-β protein expression, collagen type-IV was much more abundant in the absence of acute reperfusion, as it was the expression of its degrading protease MMP-9 and its degradation fragments. Increased MMP-9 expression could be a response to higher accumulation of collagen-IV within the injury scar, which ultimately would interfere with tissue remodeling processes in spite of a higher local MMP-9 activity. While collagen type-IV is not a growth inhibitory molecule *per se* (Tonge et al., 1997; Joosten et al., 2000), collagen meshworks in the scar can allocate other inhibitory factors that preclude vascular and neural growth signaling (Hermanns et al., 2006; Klapka and Müller, 2006), effects that may have important consequences on the endogenous mechanisms of repair. In agreement with this hypothesis, an important observation of our study is that functional outcome in the long term (7–14 days) was improved in animals that underwent early recanalization. Since the size of the lesion was though not affected, our findings strongly support that recanalization-induced enhanced vascular function in the peri-lesional regions of these animals accounts for the long-term recovery observed. These observations open the path to a more complete characterization of the effect of acute reperfusion on injury scar formation and composition, with the functional relevance that these processes may have for tissue repair and functional recovery during the chronic phase after stroke.

## Materials and Methods

All data generated or analyzed during this study are included in this published article (and its Supplementary Information Files).

### Animals

All experiments were performed in 3-month-old male C57bl/6 mice obtained from Jackson Laboratories. Mice were kept in a ventilated room with controlled temperature and with a 12-h dark/light cycle and were fed *ad libitum* with standard food and water. All experimental protocols had been approved by the Animal Welfare Committee of the Complutense University of Madrid and were in accordance to the guidelines of the Madrid Autonomous Community Government (RD 53/2013) following the European directives 86/609/CEE and 2003/65/CE. Results are reported according to ARRIVE guidelines. Details on the experimental design and number of animals used in each study are provided in Supplementary Figure 1.

### Induction of Focal Brain Ischemia

Mice were anesthetized and maintained during surgery at 1–2% isoflurane in a 30%/70% mixture of O_2_/N_2_O and body temperature was maintained at physiological levels with a heating blanket during the surgery and anesthesia recovery. Mice were exposed to focal brain ischemia by distal occlusion of the middle cerebral artery (MCAO). To study the effect of acute reperfusion, two modes of occlusion were used after randomization: permanent middle cerebral artery occlusion (pMCAO, with no reperfusion) and transient MCAO (60 min occlusion followed by reperfusion, tMCAO). MCAO was achieved through ligation of the artery with a 9-0 suture just before its bifurcation into the frontal and parietal branches (Hernández-Jiménez et al., 2013). For tMCAO, the ligation was removed after 60 min and recanalization of the artery was visually assessed. The ipsilateral common carotid artery was also occluded permanently (pMCAO) or transiently (tMCAO) prior to ligation of the MCA. Researchers were blind to the experimental groups thereafter. After surgery mice were returned to their cages with *ad libitum* access to food and water. Mice were observed until anesthesia recovery and once a day until euthanasia. When appropriate, mice were euthanized with a lethal dose of pentobarbital or an overdose of isoflurane.

### Experimental Groups

Mice were allocated in experimental groups by randomization by coin toss. A complete chart describing experimental groups, number of animals per group, dead animals (2 mice, 2%) and procedures can be found in Supplementary Figures 1, 2.

### Determination of Brain Infarct Size

Infarct size was determined at 24h by T2WI Magnetic Resonance. From 72h to 14d the injured tissue was measured by histological quantification on Nissl-stained serial sections.

### T2WI Magnetic Resonance

MRI was performed on a 1 T benchtop MRI scanner [Icon (1T-MRI); Bruker BioSpin GmbH, Ettlingen, Germany]. The system consists of a 1 T permanent magnet (without extra cooling required for the magnet). Its gradient coil provides 450 mT/m. MRI experiments consisted of a series three dimensional T2 weighted images (T2WI) used to evaluate the vascular oedema. Three-dimensional T2WI were acquired using a rapid acquisition with relaxation enhancement (RARE) technique, with a repetition time (TR) = 2350 s, RARE factor = 16, and interecho interval = 15.83 ms, resulting in an effective echo time (TE) = 95 ms, number of average = 1, the field of view (FOV) = 16 × 16 × 8 mm. The acquired matrix size was 96 × 96 × 16 and the resolution 0.167 × 0.167 × 0.500 mm. Infarcts were visualized with the Image J software. The areas of infarcted tissue, the whole ipsilateral hemisphere, and the whole contralateral hemisphere were delineated in all consecutive sections. Data were corrected for edema and expressed as % of injured hemisphere by applying the formula [(Vol_*Contra*_-Vol_*UninjuredIpsi*_)/Vol_*Contra*_] × 100.

### Histology

Cryoprotected brains were sliced using a freezing microtome (Leica) and 30 μm serial sections covering the brain were obtained. Serial sections were stained with cresyl violet and infarct volume was measured stereologically (using the software StereoInvestigator, MicroBrightField) or by ImageJ analysis at 72 h and 14 days after MCAO. The contralateral cortex and the uninjured ipsilateral cortex were traced on 10 serial sections (spaced 300 μm, covering approximately from bregma 1.3 to −2.3 mm) and volumes were calculated using the Cavalieri estimation obtaining a coefficient of error Gundersen (*m* = 1) < 0.05 in all cases. Infarct size was obtained by applying the formula for oedema correction [(Vol_*Contra*_-Vol_*UninjuredIpsi*_)/Vol_*Contra*_] × 100 and was expressed as percentage of injured ipsilateral cortex.

### Assessment of Neurological Deficits

Neuroseverity Score (James et al., 2009; Hernández-Jiménez et al., 2017) was used to measure functional deficits induced by MCAO in the mice. Motor activity score (Krupinski et al., 1993; Dimmeler et al., 1996; Kassem-Moussa and Graffagnino, 2002; Haga et al., 2003; Yamamoto et al., 2003; Chopp et al., 2007; Arkuszewski et al., 2009; Snapyan et al., 2009; Smith et al., 2017) is based on spontaneous activity, symmetry in the use of the limbs, equilibrium and coordination and sensory score (Krupinski et al., 1993; Dimmeler et al., 1996; Haga et al., 2003; Chopp et al., 2007; Thored et al., 2007; Arkuszewski et al., 2009; Snapyan et al., 2009) is based on tactile, vibrissae, visual and proprioceptive responses, that were measured in the contralateral side of the animalś; body. A minimum score of 7 reflects most severe neurological deficit and a maximum score of 21 reflects absence of deficits. The 48h measurement was taken as the basal value as we consider this time as the starting point of the angiogenesis process.

### *In vivo* Blood-Brain Barrier Permeability Assay

Mice were injected intravenously with 2% Evans Blue (2 mg/kg, Sigma) 30 min before termination at 14 days after MCAO and perfused transcardiacally with saline solution until blood was removed from the system. Contralateral and ipsilateral cortices were collected, weighted and immediately flash-frozen. Cortices were then homogenized in 250 μl 1:2 saline/50% TCA solution and homogenates were centrifuged at 10000 g for 15 min. Supernatants were collected and loaded 1:4 in 95% ethanol on a 96-well plated. Fluorescence (620 nm excitation/680 nm emission) was measured using a fluorimeter. Lowest detection level limited by the current state of the technique was 0.1 μg/ml.

### Immunofluorescence

Thirty micrometer-thickness brain serial sections were stained by free-floating immunofluorescence. Sections were incubated with primary antibodies (see Table 1 for a list of antibodies, dilutions and conditions) in blocking solution (5% Normal Serum) overnight at 4°C and then incubated with secondary antibodies conjugated to the corresponding fluorophores. For proliferation analysis, BrdU (50 mg/kg) was injected intraperitoneally once daily following the experimental designs shown in Supplementary Figure 2. For BrdU staining sections were incubated with 2N HCl at 37°C for 30 min prior to incubation with the primary antibodies. For vessel perfusion analysis, *Lycopersicon esculentum* lectin was injected intravenously 20 min prior to animal sacrifice and observed by direct FITC fluorescence under the microscope.

**TABLE 1:** Antibodies, vendors and concentrations used in immunofluorescence and western blot assays.

**Antibody/marker**	***RRID***	***Dilution***
**Immunofluorescence**		
Rabbit anti-Glut-1	Millipore Cat# 07-1401, RRID:AB_1587074	1:500
Rat anti-BrdU	Bio-Rad Cat# MCA2060T, RRID:AB_10015293	1:100
Rabbit anti-cleaved caspase-3	Cell Signaling Technology Cat# 9661, RRID:AB_2341188	1:500
FITC-lectin	Vector Laboratories Cat# FL-1171, RRID:AB_2307440	——
Isolectin B4-Alexa647		1:100
**Western blotting**		
Rabbit anti-VEGF	Santa Cruz Biotechnology Cat# sc-152, RRID:AB_2212984	1:1000
Mouse anti- Claudin-5	Thermo Fisher Scientific Cat# 35-2500, RRID:AB_2533200	1:1000
Rabbit anti-ZO-1	Innovative Research Cat# 61-7300, RRID:AB_138452	1:1000
Mouse anti-HIF-1α	Millipore Cat# MAB5382, RRID:AB_177967	1:500
Mouse anti-GFAP	Millipore Cat# IF03L, RRID:AB_2294571	1:1000
Goat anti-PDGFR-β	R and D Systems Cat# AF1042, RRID:AB_2162633	1:500
Goat anti-MMP-9	R and D Systems Cat# AF909, RRID:AB_355706	1:500
Rabbit anti-Collagen type IV	Abcam Cat# ab6586, RRID:AB_305584	1:1000

### Image Acquisition and Quantification

Regions of interest were defined as follows: infarcted cortex -where infarct rim was clearly visible in MRI images, and in Nissl (as shown in Figure 1A, by an abundant nuclei area) and immunofluorescence stainings (increased fluorescence background)- and mirroring region in the contralateral cortex (48 h, 72 h and 5 days), peri-lesional cortex (1 field immediately adjacent to the infarct rim, clearly visible by the presence of the glial scar) and the mirroring contralateral cortex (14 days). Representative images of the area comprising the infarct, the peri-infarct and the healthy tissue were taken with a 5× lens. Z-stacks used for perfusion quantification were captured using a 20x lens and analyzed volumetrically using Volocity software (Improvision). Close-up images were captured with a 40× oil-immersion lens. Vascular coverage was expressed as the sum of length of vessels (Glut-1^+^ objects) per unit of volume. Vascular tortuosity was calculated separately for each object by the ratio length/longest axis, and average vascular diameter was obtained for each vessel and expressed as a diameter size distribution for the vessel population. Cell proliferation associated to vessels was calculated by the quantification of BrdU^+^ cells with whole nuclear staining in contact with Glut-1^+^ vessels. Quantification of variations in local perfusion was performed by the analysis of the fluorescence intensity of FITC-lectin in Glut-1^+^ vessels following subtraction of background intensity. Images were captured under same exact conditions in the infarcted and contralateral cortex at sub-acute time points (72 h and 5 days) and in the ischemic boundaries and the contralateral cortex at 14 days. Data are expressed as sum of values obtained from 6 different fields of view per region of interest in each animal.

### Western Blot

Whole brain lysates were prepared from flash frozen samples by homogenization in 1x Lysis Buffer (Cell Signaling) containing protease inhibitors (Roche). Western blot membranes were obtained after PAGE-SDS and protein transfer to PVDF membranes. Membranes were incubated with primary antibodies (see Table 1 for list of antibodies, dilutions and conditions) in 5% non-fat milk in 0.2% Tween-20 TBS and HRP-conjugated secondary antibodies. Band intensity was measured by ImageJ, and data are expressed by normalization to values in the uninjured contralateral tissue due to the high variability between individuals. For that reason, the contralateral tissue was used as internal control in Figures 3C, 4C, 5A. In Figure 3D data are expressed by normalization with actin.

### Statistical Analysis

Data were expressed as mean ± SD. Data sets were tested for normality (Kolmogorov-Smirnoff test). Student’s *t*-test was used for comparison of two datasets and one- or two-way ANOVA was used for comparison of multiple datasets and variables. For comparisons against a defined value (eg.- ratio/ipsi contra = 1) a one-sample *t*-test was used. A *p*-value < 0.05 was considered statistically significant.

## Data Availability

No datasets were generated or analyzed for this study.

## Ethics Statement

All experimental protocols had been approved by the Animal Welfare Committee of the Complutense University of Madrid, and were in accordance to the guidelines of the Madrid Autonomous Community Government (RD 53/2013) following the European directives 86/609/CEE and 2003/65/CE. Results are reported according to ARRIVE guidelines.

## Author Contributions

DF-L, VD-L, MÁM, and IL designed the research. VD-L, DF-L, JG-H, AM, AG-C, SP-T, IG-Y, and AV-P performed the research. VD-L, DF-L, IG-Y, and AV-P analyzed the data. DF-L, MÁM, and IL wrote the manuscript. All authors reviewed the manuscript.

## Conflict of Interest Statement

The authors declare that the research was conducted in the absence of any commercial or financial relationships that could be construed as a potential conflict of interest.
