# Supplementary material for: Delayed Effects of Acute Reperfusion on Vascular Remodeling and Late-Phase Functional Recovery After Stroke
Source: Front Neurosci. 2019 Jul 23;13:767. doi: 10.3389/fnins.2019.00767 (PMC6664024; doi:10.3389/fnins.2019.00767)
Supplement: Supplementary file 1 [file Data_Sheet_1.docx]

Supplementary Material

# Supplementary Figures and Tables

## Supplementary Figures

**
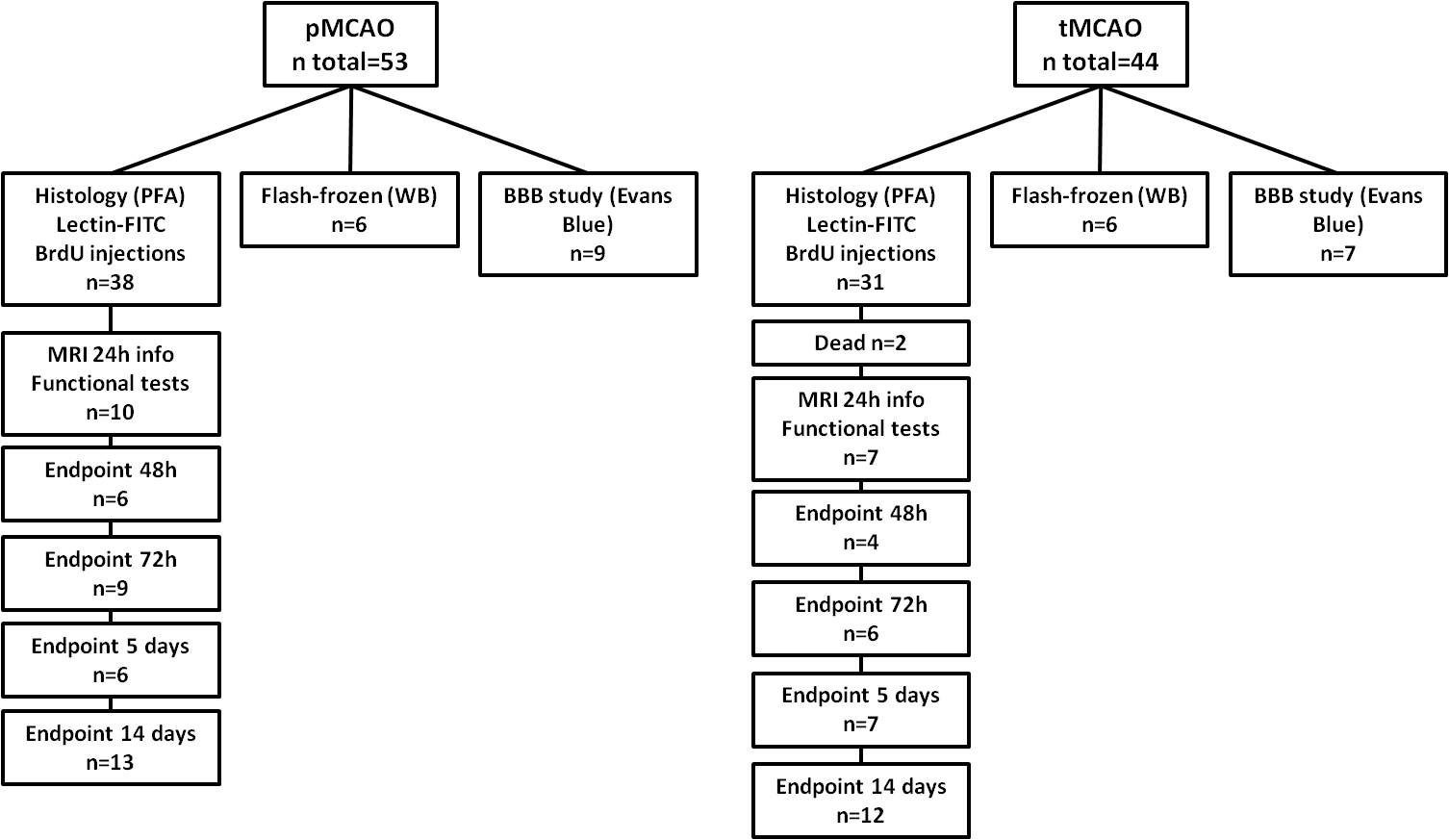
**

**Supplementary figure 1**. Distribution and numbers of animals used in the study.


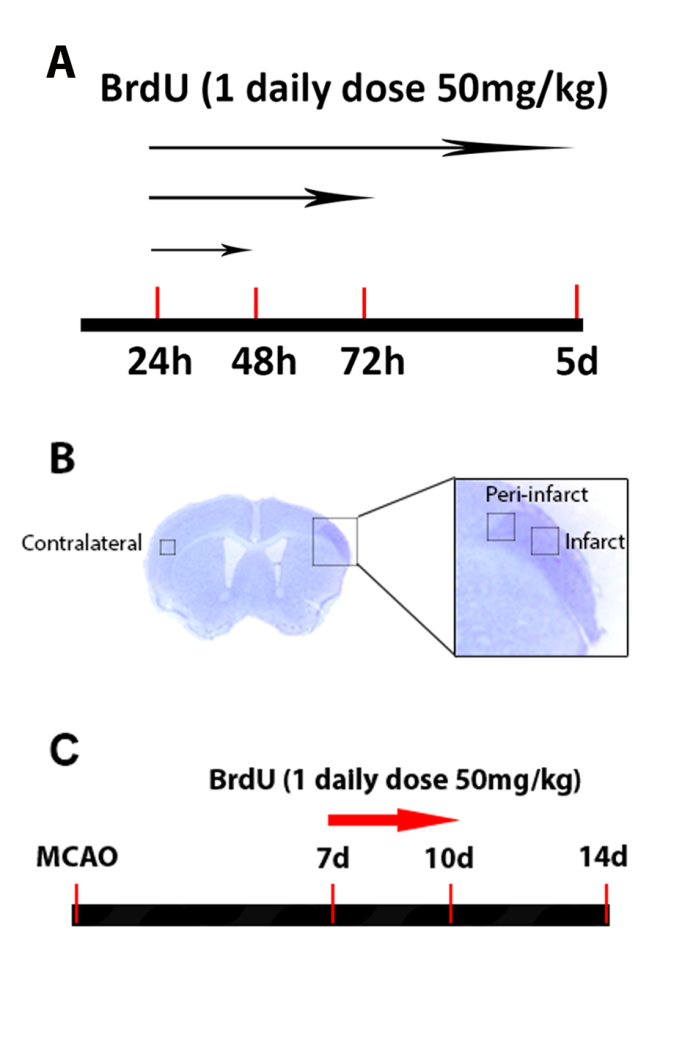


**Supplementary figure 2**. A: Protocol of sub-acute BrdU administration in mice used for endpoints at 48 hours, 72 hours and 5 days after MCAO. B: Nissl-stained micrograph depicting the regions of interest (infarct and peri-infarct) studied at 14 days after MCAO. C: Protocol of BrdU administration in mice used for endpoint at 14 days after MCAO.


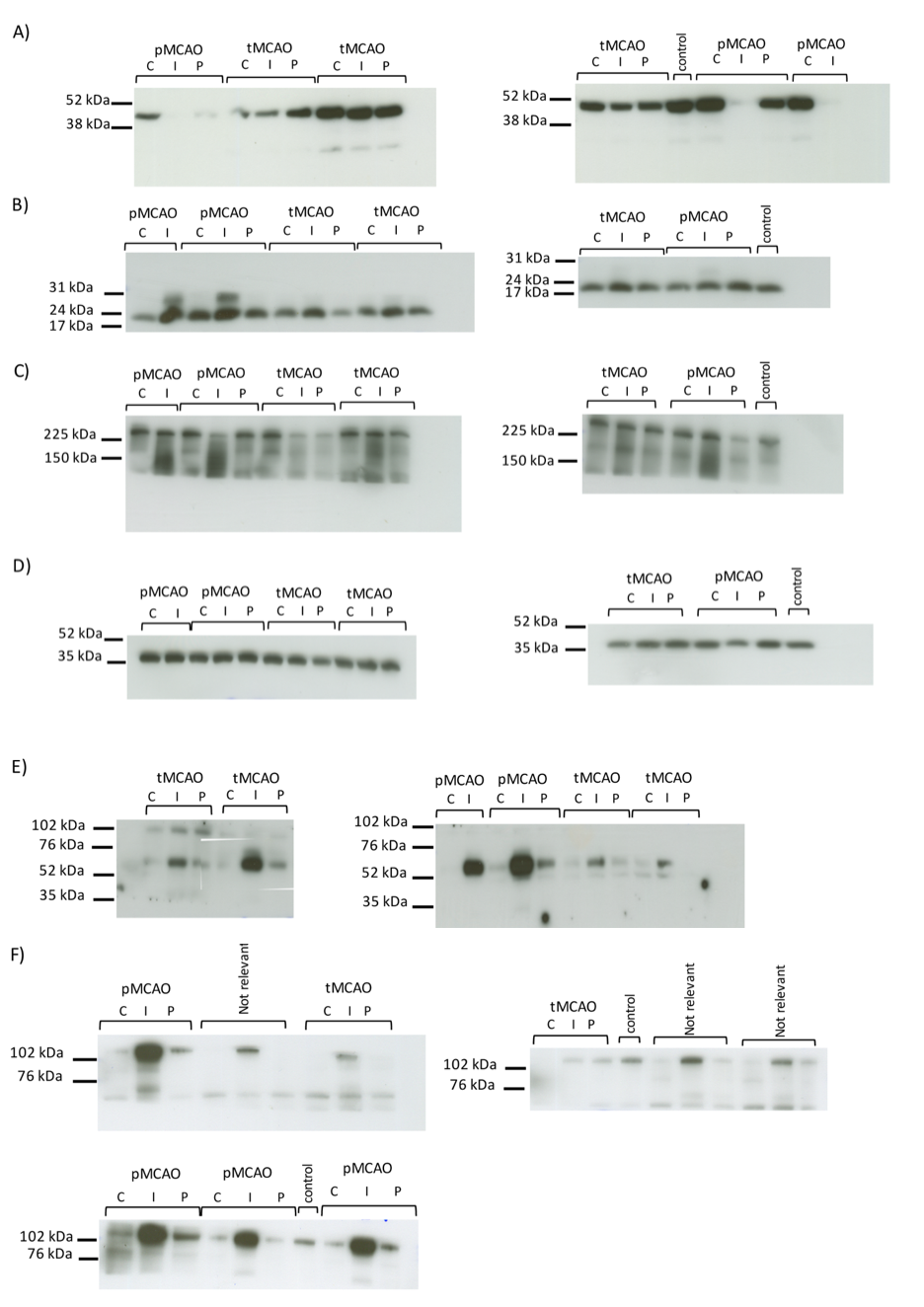


**Supplementary figure 3**. A: Original gels from VEGF-A western blot. B: Original gels from Claudin-5 western blot. C: Original gels from ZO-1 western blots. D: Original gels from Actin western blot. E: Original gels from HIF-α western blots. F: Original gels from MMP-9 western blot. G: Original gels from GFAP western blots. H: Original gels from PDGFR-β. I: Original gels from Collagen-IV western blot.
